# One sip of water with LT-4 supplementation—a key to euthyroidism in Hashimoto’s thyroiditis

**DOI:** 10.1007/s12020-024-03829-w

**Published:** 2024-04-18

**Authors:** Wolfgang J. Schnedl, Simon Michaelis, Harald Mangge, Dietmar Enko

**Affiliations:** 1 Department of Internal Medicine, Medical University of Graz, Auenbruggerplatz 15, A-8036 Graz, Austria; 2 General Internal Medicine Practice, Dr. Theodor Körnerstrasse 19b, A-8600 Bruck, Austria; 3 Institute of Clinical Chemistry and Laboratory Medicine, Hospital Hochsteiermark, Vordernberger Straße 42, A-8700 Leoben, Austria; 4 Clinical Institute of Medical and Chemical Laboratory Diagnosis, Medical University of Graz, Auenbruggerplatz 30, A-8036 Graz, Austria

**Keywords:** Hashimoto’s thyroiditis, Hypothyroidism, Thyroid peroxidase, Levothyroxine, Thyrotropin

## Abstract

**Purpose:**

Recommended pharmacotherapy for hypothyroidism in Hashimoto’s thyroiditis (HT) is oral supplementation with levothyroxine (LT-4). However, serum thyrotropin (TSH) levels within normal range are not consistently achieved with LT-4 medication.

**Patients and methods:**

We report on 35 HT patients with LT-4 therapy in this retrospective evaluation. In general, we recommend that a maximum of two sips of water, which would then amount to < 50 mL, be ingested at the same time as LT-4. We report on follow up examinations measuring TSH and antibodies against thyroid peroxidase (TPOAb) after 6 months to five years.

**Results:**

After median time of 643 days (range 98-1825) we found in 35 HT patients a statistical significant reduction of serum TSH (p < 0.001) and TPOAb (p = 0.006). The patients median body weight was 71 kg (range 48–98) and a daily LT-4 dosage was used with median 69.1 µg (range 25–150). This results in a daily LT-4 dose of median 1.01 µg/kg bodyweight (range 0.3–2.3).

**Conclusions:**

The reduction of water ingestion to a maximum of two sips, which is <50 mL, combined with LT-4 supplementation helps to achieve euthyroidism in HT. In addition, it reduces the L-T4 medication dosage needed to lower TSH serum levels and decreases TPO antibodies in HT.

## Introduction

Hashimoto’s thyroiditis (HT), also known as chronic lymphocytic thyroiditis, is the most common autoimmune disease [1]. The pathophysiology of autoimmune thyroid syndromes, including Graves’ and Basedow’s disease, is believed to result from a combination of genetic and environmental factors, as well as other immune disorders. Thyroid peroxidase (TPO) antibody levels, the major autoantigen in HT, correlate with the degree of lymphocytic infiltration in thyroid tissue. Subsequently, serum measurements of TPO antibody titers are clinically valuable in the diagnosis of HT. For HT caused hypothyroidism synthetic levothyroxine (L-T4) remains the mainstay of treatment [2, 3].

In recent decades, thyroid function assessment has primarily relied on measuring the circulating level of thyroid stimulating hormone (TSH). It has been proposed to switch from measuring the TSH level to the level of thyroid hormones [4]. However, in clinical routine serum TSH is used as the most important laboratory parameter [5], but suggested TSH levels within the normal range are not consistently achieved with LT-4 supplementation [6]. In line with this, best practice guidelines for the management of hypothyroidism have been published by international societies [2, 3]. These recommendations mention the avoidance of a number of factors which may interfere with the gastrointestinal L-T4 absorption causing insufficient L-T4 treatment. A possible factor that impairs the absorption of L-T4 is a decrease in gastric acid and an increase in pH [7]. Generally, the amount of water to be swallowed with L-T4 tablets is not specified [2, 3]. In this retrospective evaluation we show that reducing water intake to a maximum of two sips, combined with LT-4 supplementation can help to achieve euthyroidism. In addition, it reduces the dose of L-T4 treatment needed to achieve TSH values within the normal range and reduces TPO antibody titers in HT.

## Methods

This pilot evaluation retrospectively included 35 consecutive patients with Hashimoto’s disease, all of whom were white. Blood samples were taken in the morning, at the first presentation and at controls, after an overnight fast [>12 h] and without taking the L-T4 medication. Patients were interviewed in detail about their water intake, dietary habits and the timing of their L-T4 intake versus breakfast. In 9 of 35 HT patients symptoms of subclinical hypothyroidism included globous sensation, impaired swallowing, chronic fatigue, nervousness and mood swings. Non-compliant patients who approved that they were not thoroughly following the recommendations for LT-4 supplementation, those with histologically proven thyroid cancer, or those with Basedow’s and Graves’ diseases were excluded.

Serum thyrotropin was determined using the chemo luminescence immunoassay Atellica IM Analyzer (Siemens Healthcare GmbH, Erlangen, Germany). Antibodies against thyroid peroxidase (TPO) and thyrotropin receptor antibodies (TSH-R-Ab) were measured with chemo luminescence using the Alinity I Immunology Analyzer (Abbott, Chicago, Illinois, USA).

Until subsequent controls, we recommended that L-T4 tablets be taken on an empty stomach in the morning. The medication LT-4 should be taken at least 30 min before breakfast, preferably 45 min, with no more than two sips of water to aid in swallowing. It has been suggested that all other medical treatments should be taken separately from L-T4, and at the earliest with breakfast. The patients were instructed not to take any liquids other than water. In particular, they were advised not to drink any fruit juices, drinks or milk in combination with LT-4. And they were instructed not to smoke during those 30 min before breakfast. Between 6 months and 5 years after initial presentation, TSH, TPO antibodies, and TSH-R-Ab were checked. Combined L-T4 and liothyronine (L-T3) therapy was routinely discouraged. If used, it was discontinued at first presentation.

**Fig. 1:**
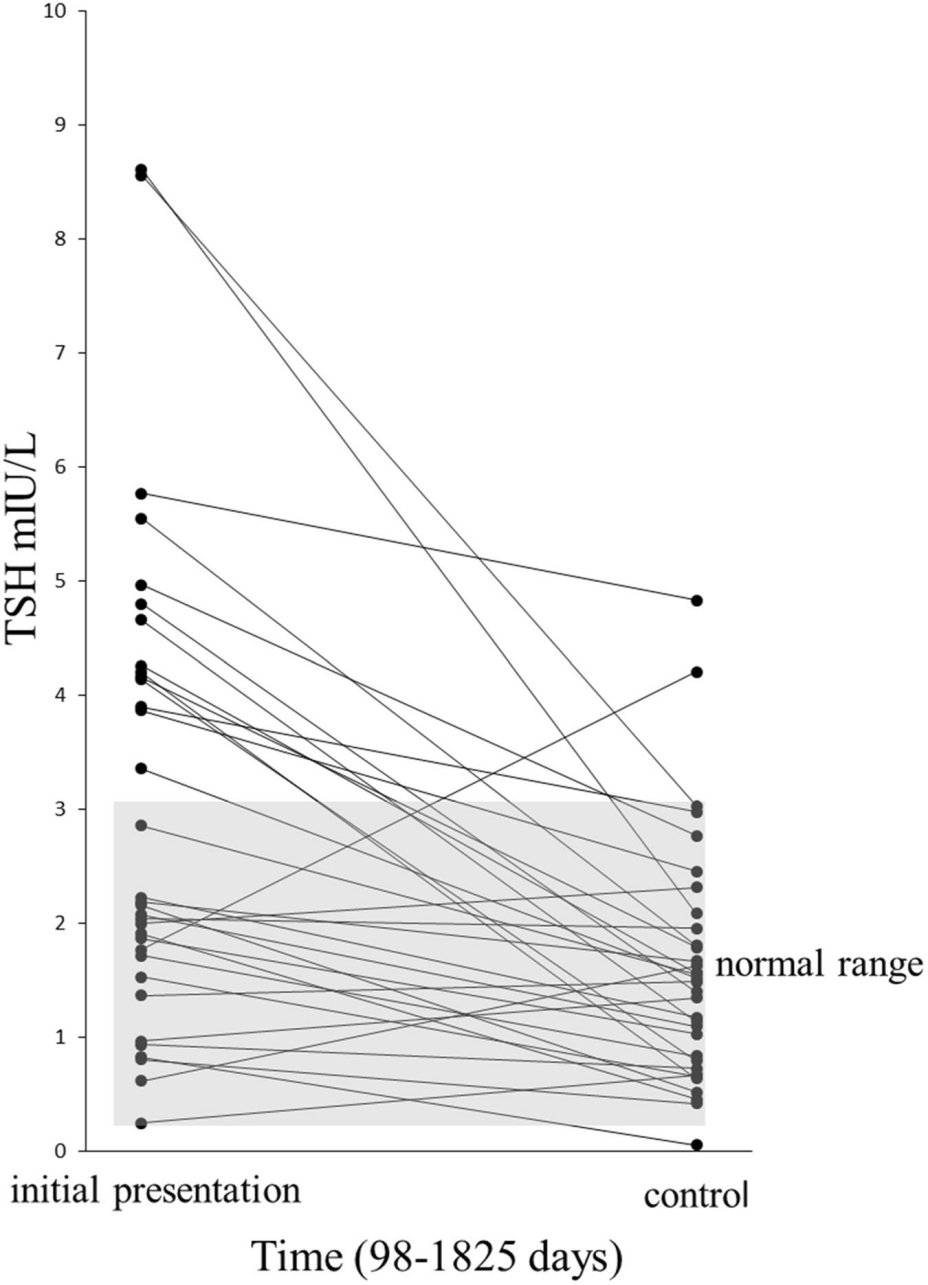
Comparison of TSH measurements showed statistically a significant decrease (p < 0.001) between initial measurement and controls in HT patients

**Fig. 2:**
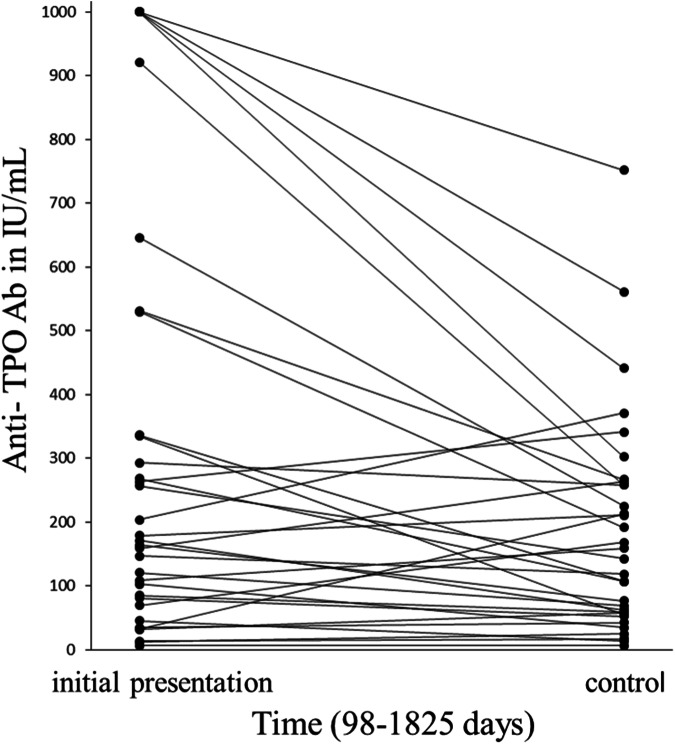
Comparison of TPO-Ab measurements showed a statistically significant difference (p = 0.006) between initial measurement and controls in HT patients

In this study, we performed a longitudinal observation of the treatment efficacy. For each patient, the comparison was made between the baseline and follow-up data. Statistical analyses were performed using SPSS 29.0 (SPSS Inc, Chicago, IL, USA). As a nonparametric statistical test for paired comparisons of TSH and TPO-Ab measurements, the Wilcoxon signed rank test demonstrated statistical significant difference from the initial measurement to the second test in HT patients (Figs. 1, 2). Data distribution was assessed by the Shapiro-Wilk test with quantitative parameters summarized as median and interquartile range (IQR) according to distribution. The Spearman rho rank correlation was used as a nonparametric measure for correlation of LT-4 dosage, age, and body weight. Statistical significance was defined as a p value of less than 0.05.

## Results

During this retrospective study 35 HT patients with a median age of 46.1 years (age range 16.9-59.1) were evaluated. Of these patients, 32 were female and 3 were male. At presentation serum TSH was median 3.05 U/mL (0.25–8.61), TPO-Ab measurements were elevated with median 328 U/mL (range 7–1000) indicating HIT. After median time of 643 days from first presentation to controls (range 98–1825) the TSH was median 1.61 U/mL (range 0.06–4.83), TPO-Ab measurements were median 198 U/mL (range 7–752). To rule out Graves’ and Basedow’s disease, TSH-R-Ab levels were checked in all patients at presentation and during follow-up visits, and found to be within the normal range. For 35 HT patients we found a body weight of median 71 kg (range 48–98) and a daily LT-4 dosage used with median 69.1 µg (range 25–150). This results in a daily LT-4 dose of median 1.01 µg/kg bodyweight (range 0.3–2.3). The comparison between the presentation and control data for TSH and TPO-Ab revealed a statistical difference, p < 0.001 and p = 0.006, respectively (Figs. 1, 2). There is no significant correlation between LT-4 dosage and age (p = 0.2) or body weight (p = 0.061).

## Discussion

Mostly asymptomatic HT silently interferes with thyroid function and hormone production [1]. Subclinical hypothyroidism occurs with TSH values < 10 U/mL (normal range 0.2 – 3.8) and the autoimmune process may cause hypothyroidism related symptoms including e.g. globous sensation and impaired swallowing. Some patients have ongoing symptoms even though their TSH level is measured within the normal range [8]. In our patients who presented with complaints related to hypothyroidism, the symptoms improved within a week of following the < 50 ml water recommendations for the use of LT-4.

It is unclear whether thyroid function is normal due to inter- and intra-individual differences in thyroid parameters, transient variations, age-related differences, and ethnic variations. The thyroid function of each patient needs be evaluated based on the individual clinical picture [9]. The effect of body weight on LT-4 requirements is currently being intensively studied [10]. However, bariatric surgery may affect the absorption of exogenous medications, including LT-4, due to its impact on the gastrointestinal tract [11, 12]. In our study there was no significant correlation found between age and body weight with LT-4 dosage. Several evaluations for LT-4 absorption have been developed for patients with refractory hypothyroidism to define a LT-4 malabsorption [13]. *Helicobacter pylori* infection can alter gastric acid secretion, which may impair LT-4 drug absorption [14]. It has been demonstrated that an increased stomach pH can affect the pharmacokinetics of ingested LT-4 [15]. To optimize the administration of LT4, various formulations were considered, including intravenous, intramuscular, and rectal routes [16]. Non-tablet formulations to improve the absorption of L-T4 are being investigated [17], including liquid and soft gel capsules [18]. A recent review described a list of pharmaceutical, pathophysiological, and patient behavioral factors that may influence LT-4 absorption [19]. Nonetheless, further clinical and experimental studies are needed to determine whether the reduction in water intake is beneficial when taking LT-4 medications. We cannot exclude the possibility of selection bias in our single-center experience.

In general, LT-4 is a safe and effective hormone replacement for conditions of hypothyroidism including autoimmune thyroiditis, partial or total thyroidectomy, and after radioiodine treatment [20]. However, LT-4 has a narrow therapeutic window and up to 50% of patients fail to maintain a desired serum TSH level within the normal range [8]. Patients with TSH values above the normal range should be reassessed to ensure that L-T4 is being administered correctly [21] and that all known factors interfering with L-T4 absorption have been addressed [22, 23]. Despite this, a number of patients does not respond properly to LT-4 and require higher doses, necessitating ongoing monitoring. Unfortunately, non-compliance also is common and should be considered.

Based on clinical experience, we generally recommend a maximum of two sips of water, which would be < 50 ml, at the same time as LT-4. Normal stomach acidity is maintained at a pH between 1.0 and 3.0, which is essential for the digestion and absorption of medications [24]. This is supported by the increased dosage of LT-4 required in patients with gastric *Helicobacter pylori* infection, chronic atrophic gastritis, gastroparesis, or concomitant treatment with drugs, including proton pump inhibitors which reduce gastric acid production [25]. The time between ingestion and plasma appearance suggests that LT-4 is mainly absorbed in the jejunum and ileum. The absorption in the stomach and the duodenum is described as being insignificant [26]. Nevertheless, the stomach plays a key role for efficient absorption [21].

The stomach becomes hypo- and/or achlorhydric when acid secretion is impaired or gastric acid is diluted. Increase of gastric pH is a significant source of variability in the absorption of orally administered drugs [27]. In a hypo- and/or achlorhydric gastric environment, sodium levothyroxine salt is incompletely dissolved, resulting in ineffective intestinal absorption [7]. In general, drinking 250 mL or more of water dilutes the gastric acid and gastric acid insufficiency is manifested by reduced buffering capacity, chloride ion concentration, osmolality, low surface tension in the stomach, and by increased pH in the upper intestine [24]. Therefore, slow and incomplete dissolution of drugs under high gastric pH conditions may ultimately result in a loss of efficacy [19].

Before first presentation in our outpatient setting, the patients described the amount of water they swallowed simultaneously with LT-4 as ranging from the smallest of approximately 100 mL to a glass of 250 mL. The manufacturer’s package inserts suggest the use of half a glass (~125 mL) to one glass of water (~250 mL) with the ingestion of LT-4 [28, 29]. Clinical guidelines recommend that LT-4 should be taken with water, but do not specify the quantity of water required [2, 3].

An estimate for sipping of water is defined as approximately 25 mL per sip for men and 20 mL per sip for women [30]. However, we generally recommend reducing the water to less than 50 mL, or a maximum of two sips, when swallowing the LT-4 tablet. This helped to lower TSH levels into the normal range and decreased TPO-Abs for up to 5 years (Figs. 1, 2).

Several calculation methods have been developed to determine individual LT-4 dose requirements. These methods include estimating the dosage based on total body weight, body mass index (BMI), ideal body weight, and lean body mass. It has been reported that patients with residual endogenous thyroid function, such as those with autoimmune thyroiditis, require a dose of LT-4 with 1.6 µg/kg/day [6]. In our study of patients with HT, we found that a daily dose of LT-4 at 1.01 µg/kg/day was effective. This dosage is evidently lower than the previously described quantity.

## Conclusion

It has been demonstrated that reducing water intake to less than 50 mL, or a maximum of two sips, in combination with L-T4 intake, reduces the required dosage of L-T4 to lower TSH levels to euthyroidism in patients with HT. Additionally, a significant decrease in TPO-Abs was observed in patients with Hashimoto’s disease.

## Data Availability

The data that support the findings of this study are available from the corresponding author upon reasonable request.

## Abbreviations

HT: Hashimoto’s thyroiditis
LT-4: Levothyroxine
TPOAb: Thyroid peroxidase antibodies
TSH-R-Ab: thyrotropin receptor antibodies
L-T3: liothyronine;
